# Obstetric penicillin allergy evaluations

**DOI:** 10.1016/j.jacig.2024.100376

**Published:** 2024-11-26

**Authors:** Lakshmi G. Nair, S. Shahzad Mustafa, Allison Ramsey

**Affiliations:** a Division of Allergy and Clinical Immunology, National Jewish Health, Denver; b Division of Allergy, Immunology, Rheumatology, Rochester Regional Health; c Division of Allergy, Immunology, Rheumatology, University of Rochester

**Keywords:** Penicillin allergy, drug allergy

## Abstract

**Background:**

Penicillin allergy is reported in 5% to 15% of the world population, with 3% to 10% of pregnant women reporting the same. However, more than 90% of these patients can tolerate penicillin after appropriate evaluation. Penicillin is indicated for various issues that arise in pregnancy, and a history of allergy can have negative individual and public health consequences.

**Objective:**

Our aim was to prospectively evaluate the feasibility, safety, and select obstetric outcomes of obstetric penicillin allergy evaluations arranged through a direct referral phone line from obstetric practices to an employed allergy/immunology practice.

**Methods:**

Patients were referred via direct phone line for evaluation during their antenatal visits between May 2019 and May 2022. Patients underwent skin prick testing, and those with a negative penicillin skin testing (PST) result were subjected to amoxicillin challenge. In select cases of patients with a low-risk history, direct oral challenge was performed. Data were analyzed using descriptive statistics.

**Results:**

Of the 324 patients referred between May 2019 and May 2022, a total of 251 (77.5%) presented for in-office evaluations. Of those 251 patients, 239 (95.2%) underwent PST followed by oral challenge if the PST result was negative; 12 patients (4.8%) underwent direct challenge without skin testing, and all of them passed the challenge. Of the patients undergoing PST, 230 (97.2%) had a negative result and 229 tolerated subsequent oral amoxicillin doses, with 1 patient experiencing a delayed reaction to the amoxicillin. The group of patients who presented for evaluation included more people living in ZIP codes described as being of high socioeconomic status than in the no-show group (73.7% vs 63.3%).

**Conclusion:**

To our knowledge, ours is the largest study to date to demonstrate the safety and feasibility of a phone line for obstetric penicillin allergy referrals. We demonstrate a better show rate than previous analyses, with most of the patients presenting for evaluation being successfully delabeled.

## Introduction

Penicillin allergy is reported in 5% to 15% of the world population, with 3% to 10% of pregnant women reporting the same. However, more than 90% of these patients can tolerate penicillin after appropriate evaluation.^1^ Previous studies have shown that carrying a diagnosis of penicillin allergy can lead to negative individual and public health consequences in the nonpregnant population, as alternative antibiotics may have more side effects, demonstrate less efficacy, and be more costly. In pregnancy, β-lactam antibiotics are the first line for group B *Streptococcus* prophylaxis and preoperative prophylaxis for caesarean sections, but they may also be needed for other conditions, including preterm rupture of membranes, listeriosis, syphilis, and chorioamnionitis. Almost one-third of pregnant patients require penicillin in pregnancy for prophylaxis or treatment of various conditions.^1^

We prospectively evaluated the feasibility, safety, and select obstetric outcomes of obstetric penicillin allergy evaluations arranged through a direct referral phone line from obstetric practices to an employed allergy and immunology practice. Patients were referred via direct phone line for an allergy and immunology evaluation during any of their antenatal visits between May 2019 and May 2022. Referrals were made regardless of pregnancy trimester. Patients were scheduled with 2 allergy and immunology physicians on the basis of allergy and immunology nurse discretion. Patients underwent penicillin skin testing (PST) and intradermal testing using the major determinant of penicillin (penicilloyl polylysine [Pre-Pen, ALK, Round Rock, Tex]) and penicillin G (10,000 U/mL), histamine (6 mg/mL for prick and 0.02 mg/mL for intradermal injection), and a saline negative control. Patients with a negative PST result were given a 400-mg amoxicillin challenge and monitored for 30 minutes. In select cases, direct oral challenge was performed in patients with a low-risk history, such as family history of penicillin allergy or history suggestive of an intolerance. Demographic information, including age, race, weeks of gestation, gravida status, ZIP code, historical reaction, and time since reaction, were recorded, as were the outcomes of the testing. Subsequent chart review was conducted for enrolled patients to determine the mode of delivery and any neonatal complications. Data were analyzed using descriptive statistics. This study was approved by the Rochester Regional Health Institutional Review Board.

## Results and discussion

Of the 324 patients referred between May 2019 and May 2022, a total of 251 (77.5%) presented for in-office evaluations. Their median age was 30 years (interquartile range [IQR] = 27-35 years), and their median time since the reaction was 25 years (IQR = 16-32 years). Of the 251 patients presenting for in-office evaluation, 221 (88.04%) had a history of skin reaction, 13 (5.18%) had a history of angioedema, 14 (5.58%) had a history of anaphylaxis, and 3 (1.2%) reported only a family history. Of the patients evaluated, 70.5% reported 1 drug allergy, with the others reporting more than 1 drug allergy. Of the 251 patients evaluated, 61 (24.3%) had a history of group B streptococcal infection. The baseline characteristics of the patients presenting for evaluation versus those of the patients who did not are detailed in Tables I and II. Of the 251 patients, 239 (95.2%) underwent PST followed by oral challenge if the PST result was negative; 12 patients (4.8%) underwent direct challenge without skin testing owing to a family history of penicillin allergy only or a personal history of nausea, vomiting, or diarrhea with penicillin. All of the patients (100%) who underwent direct oral challenge, passed the challenge, and penicillin allergy was removed from their charts. Of the patients undergoing PST, 230 (97.2%) had a negative result and 229 tolerated subsequent oral amoxicillin doses, with 1 patient experiencing a delayed reaction to the amoxicillin. Five patients (2.1% in total) had a positive skin testing result: 2 of the 5 had a positive reaction to penicillin G and 3 of the 5 had a positive reaction to penicilloyl polylysine (Pre-pen). In addition, 4 patients (1.7%) had an indeterminate skin testing result and continued to avoid penicillin-based antibiotics. Compared with the no-show group, the group of patients who presented for evaluation were more likely to be White and live in ZIP codes found to be characterized by residents of high socioeconomic status (73.7% vs 61.6%). For data regarding mode of delivery in the 2 groups, please refer to Tables I and II. The median length of stay of the neonates after delivery was 2.78 days (IQR = 2.27-3.54 days). Of the 148 neonates, 5 (3.38%) required antibiotics after delivery. Otherwise, no significant differences between the 2 groups were noted.

**Table I:** Baseline characteristic of the participants who underwent outpatient evaluation

Characteristic	Value (N = 251)
Age (y), median (IQR)	30 (15-34)
Race, no. (%)	
Black	26 (10.4%)
White	194 (77.3%)
Asian	0
Other	29 (11.6%)
Unknown	2 (0.8%)
Gestational age, no. (%)	
First trimester	9 (3.6%)
Second trimester	129 (51.4%)
Third trimester	110 (43.8%)
Unknown	3 (1.1%)
Mode of delivery, no. (%)	
Cesarean section	72 (33.1%)
Vaginal	137 (54.6%)
Abortion	2 (0.8%)
Unknown	40 (15.9%)
Time since last reaction (y), median (IQR)	25 (16-32)
Historical type of reaction, no. (%)	
Hives/itching	102 (40.6%)
Rash	83 (33.1%)
Anaphylaxis	11 (4.4%)
Swelling/angioedema	14 (5.6%)
Family history	3 (1.2%)
Skin peeling	1 (0.4%)
Unknown	32 (12.7%)
Other	5 (2%)
Drug allergies, including penicillin, no. (%)	
0	1 (0.4%)
1	176 (70.1%)
2	49 (19.5%)
3	13 (5.2%)
≥4	12 (4.8%)
Socioeconomic status	
High	185 (73.7%)
Low	66 (26.3%)

**Table II:** Baseline characteristics of the participants who did not undergo outpatient evaluation

Characteristic	Value (n = 73)
Age (y), median (IQR)	29 (23-34)
Race, no. (%)	
Black	17 (23.3%)
White	40 (54.8%)
Asian	0
Other	16 (21.9%)
Gestational age, no. (%)	
First trimester	1 (1.4%)
Third trimester	4 (5.5%)
Unknown	68 (93.1%)
Mode of delivery, no. (%)	
Cesarian section	16 (21.9%)
Vaginal	41 (56.2%)
Abortion	2 (2.7%)
Unknown	14 (19.2%)
Historical type of reaction, no. (%)	
Hives/itching	26 (35.6%)
Rash	10 (13.7%)
Anaphylaxis	4 (5.5%)
Swelling/angioedema	3 (4.1%)
Unknown	30 (41.1%)
Drug allergies, including penicillin, no. (%)	
1	40 (61.5%)
2	17 (26.2%)
3	2 (3%)
≥4	6 (9.1%)
Unknown	8 (12.3%)
Socioeconomic status	
High	45 (61.6%)
Low	28 (38.4%)

We have thus demonstrated the safety and feasibility of a direct phone line for outpatient penicillin allergy referrals in the obstetric patient population. To our knowledge, our study examined the largest reported cohort of pregnant women undergoing penicillin allergy testing. Most of the patients (77.5%) presented for evaluation, and 96% of those patients were successfully delabeled. There were no significant differences between those who presented for a penicillin allergy evaluation and those who did not, aside from a possible lower socioeconomic level of those in the latter group.

A unique aspect of our analysis is the relatively high rate of presentation for evaluation compared with that in other reports in the literature.^2^^,^^3^ Wolfson et al reported their experience with electronic consults conducted before in-person obstetric appointments; they stated that 222 of 363 of patients evaluated via e-consult (61%) presented for their in-person appointment.^2^ Another analysis by Stephen et al reported a dedicated penicillin allergy referral pathway with centralized scheduling, demonstrated that 69 of 102 patients (68%) presented for evaluation. Our higher rate of presentation was likely due to our approach of using a dedicated phone line, thus allowing patients to be scheduled while still physically present at their obstetric appointment.

There are emerging data on direct challenges in pregnant women with low-risk reaction histories. A Canadian analysis by Mak et al reported on the safety of such challenge in 203 patients.^4^ Our study had a small number of patients undergoing direct challenge. However, given the safety demonstrated in our approach, along with the data from Mak et al and the accruing data in the nonpregnant population,^5^ it is reasonable to conclude that direct challenge can be used more frequently in the pregnant population.

Our analysis raises a question about whether socioeconomic status and race affect whether a patient presents for penicillin allergy evaluation. Lower socioeconomic status is associated with poorer pregnancy outcomes,^6^ with complex factors underlying these findings. Strategies to overcome this finding deserve more study.

The main weakness of our analysis is the relatively low number of patients, which limited demonstration of differences in pregnancy outcomes and neonatal outcomes between the 2 groups. Our approach may also not be generalizable to different practice settings.

We are, to our knowledge, the largest study to date to demonstrate the safety and feasibility of a dedicated phone line with a dedicated nurse who corresponds with the referring practitioner office’s referrals related to obstetric penicillin allergy. We demonstrate a better show rate than shown by previous analyses, with most of our patients being successfully delabeled.

**Clinical implications**

A direct referral phone line may improve presentation rates for obstetric penicillin allergy evaluation. The majority of patients who presented for evaluation were safely delabeled. Direct oral challenge is safe in pregnant patients with a low-risk reaction history.

## Disclosure statement

Disclosure of potential conflict of interest: S. S. Mustafa serves on the speakers bureau for Sanofi/Regeneron, AstraZeneca, GlaxoSmithKline, CSL Behring and has a grant from Takeda. A. Ramsey serves on the speakers bureau for Sanofi/Regeneron, AstraZeneca, and GlaxoSmithKline. The remaining author declares no relevant conflicts of interest.
